# Heparan sulfate proteoglycan‐mediated dynamin‐dependent transport of neural stem cell exosomes in an in vitro blood–brain barrier model

**DOI:** 10.1111/ejn.14974

**Published:** 2020-09-30

**Authors:** Bhagyashree S. Joshi, Inge S. Zuhorn

**Affiliations:** ^1^ Department of Biomedical Engineering University of Groningen University Medical Center Groningen Groningen The Netherlands

**Keywords:** blood–brain barrier, cargo, endothelial cell, exosomes, extracellular vesicles, heparan sulfate proteoglycans, nanocarriers, transcytosis

## Abstract

Drug delivery to the brain is greatly hampered by the presence of the blood–brain barrier (BBB) which tightly regulates the passage of molecules from blood to brain and vice versa. Nanocarriers, in which drugs can be encapsulated, can move across the blood–brain barrier (BBB) via the process of transcytosis, thus showing promise to improve drug delivery to the brain. Here, we demonstrate the use of natural nanovesicles, that is, exosomes, derived from C17.2 neural stem cells (NSCs) to efficiently carry a protein cargo across an in vitro BBB model consisting of human brain microvascular endothelial cells. We show that the exosomes are primarily taken up in brain endothelial cells via endocytosis, while heparan sulfate proteoglycans (HSPGs) act as receptors. Taken together, our data support the view that NSC exosomes may act as biological nanocarriers for efficient passage across the BBB. Nanomedicines that target HSPGs may improve their binding to brain endothelial cells and, possibly, show subsequent transcytosis across the BBB.

## Graphical abstract

Neural stem cell‐derived exosomes interact with heparan sulfate proteoglycans on endothelial cells at the apical (blood) side and are taken up by dynamin dependent endocytosis. II. Exosomes are transported across the endothelial cell and reach the basal (brain) side.

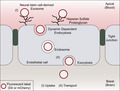

**Abbreviations**
BBB: blood–brain barrier
DLS: dynamic light scattering
DMA: dimethylamiloride
Dyn: dynasore
EBM‐2: endothelial basal medium 2
FBS: fetal bovine serum
hCMEC: human cerebral microvascular endothelial cells
HS: heparan sulfate
HSase: heparinase III
HSPGs: heparan sulfate proteoglycans
MVB: multivesicular body
NSCs: neural stem cells
SDC2: syndecan‐2
XP: XPack

## 1 INTRODUCTION

Delivery of therapeutics to the brain remains a major challenge to date due to the presence of the blood–brain barrier (BBB) which restricts the entry of therapeutics from the blood to the brain (Tang et al., 2019). The BBB consists of a layer of tightly connected endothelial cells that lines the brain capillaries and actively regulates the transport of molecules to the brain (Chow & Gu, 2015). The main function of the BBB is to preserve brain homeostasis and protect the brain from harmful substances and unwanted immune responses (Andreone et al., 2015; Banks, 2016; Obermeier et al., 2013). The tight junctions between adjacent endothelial cells limit the paracellular diffusion of hydrophilic molecules across the BBB (Chow & Gu, 2015), while lipophilic molecules that enter the BBB via passive diffusion (Abbott & Romero, 1996; Begley & Brightman, 2003) are transported back to the blood by multidrug resistance proteins present in the plasma membrane of the endothelial cells (Zhang et al., 2004). This restrictive nature of the BBB hampers the development of treatments for brain disorders.

Stem cells that are engineered to express therapeutic biomolecules have emerged as a promising drug delivery strategy in recent years (Aboody et al., 2008; Lee et al., 2000). NSCs, in particular, have shown to target sites of neurodegeneration and cerebral ischemia when administered intracerebrally and intracerebroventricularly (Bjugstad et al., 2005; Kelly et al., 2004) (Aboody et al., 2008; Dickson et al., 2007). Additionally, NSCs show the inherent property to transmigrate across the BBB (Diaz‐Coranguez et al., 2013). Although effective in the delivery of therapeutics, inflammation due to allogenic responses and differentiation into unwanted specialized cells in response to the microenvironment complicate the use of stem cells as drug delivery vehicles (Aleynik et al., 2014). Moreover, only 1% of intravenously injected stem cells reach the brain, while the majority ends up in other organs, mainly liver, lungs, and kidneys (Barbash et al., 2003). Thus, an approach that increases the brain‐homing capacity of stem cells and avoids their potential harmful side effects is needed.

Nanoscale vesicles known as exosomes are released by cells to communicate with other cells at nearby and distant locations. Exosomes mirror the composition of their cells of origin and selectively target cells with a similar phenotype (Antimisiaris et al., 2018; Hoshino et al., 2015; Wiklander et al., 2015). Therefore, we hypothesized that exosomes derived from NSCs would show the capacity to cross the BBB, showing organotropism toward the brain. Additionally, exosomes engineered to contain therapeutic cargo could act as drug delivery vehicles overcoming the disadvantages of the use of whole stem cells. In this study, we show that exosomes derived from C17.2 NSCs efficiently cross an in vitro BBB without hampering the endothelial cell monolayer integrity. These data are in line with previous findings that exosomes derived from cell types such as dendritic cells (Alvarez‐Erviti et al., 2011; Cooper et al., 2014), brain endothelial cells (Yang et al., 2017), macrophages (Haney et al., 2015; Yuan et al., 2017), and mesenchymal stromal cells (Zhang et al., 2015) show transport across the BBB in vitro and in vivo (Ha et al., 2016; Khan et al., 2018; Niu et al., 2019). In addition, we demonstrate that NSC exosomes interact with brain endothelial cells through HSPGs and that dynamin‐dependent endocytosis plays a role in the uptake of exosomes into these cells. Furthermore, we genetically engineered NSCs to package a fluorescent protein, that is, mCherry, in the interior of exosomes. Following incubation of the in vitro BBB with apically added mCherry‐loaded exosomes, mCherry was detected at the basolateral side of the BBB, indicating that NSC‐derived exosomes effectively carry their cargo across an in vitro BBB. These findings encourage the design of NSC‐derived exosomes for drug delivery to the brain.

## 2 Methods

### 2.1 Plasmids

mCherry cDNA was amplified from pcDNA3.1 SP‐His‐mCherry‐HRP‐VhHGFP FLIPPER‐body vector (de Beer et al., 2018) (a gift from Ben Giepmans; addgene plasmid #112157; http://n2t/addgene:112157; RRID:Addgene_112157) and inserted in XPack (XP) CMV‐XP‐MCS‐EF1‐Puro Cloning Lentivector (purchased from SBI biosciences; XPAK510PA‐1) between XhoI and EcoRI to generate pCMV‐XP‐mCherry‐EF1‐Puro.

### 2.2 Antibodies and reagents

For immunoblotting, primary antibodies against mCherry (rabbit; Abcam ab167453; 1:1,000), CD9 (rabbit; Abcam ab92726; 1:1,000), β‐actin (rabbit; Abcam ab8227; 1:2000), and TSG101 (mouse, Genetex GTX70255, 1:1,000) were used. The following Odyssey secondary antibodies were used: anti‐mouse, and ‐rabbit antibodies (Li‐COR, LI 926‐68070, LI 925‐32211) at 1:5,000 dilution for the final detection. For immunocytochemistry, Syndecan‐2 (SDC2, rabbit; Santa Cruz sc‐15348; 1:50) was used followed by staining with secondary antibodies conjugated with Alexa 488 (goat anti‐rabbit; Invitrogen A‐10680; 1:500).

### 2.3 Cell culture

Human cerebral microvascular endothelial hCMEC/D3 cells were cultured in endothelial basal medium 2 (EBM‐2) (Lonza CC‐3156) supplemented with 1.4 μM hydrocortisone (Sigma‐Aldrich H0888), 1 ng/ml human basic fibroblast growth factor (Peprotech 100‐18B), 5 μg/ml ascorbic acid (Sigma‐Aldrich A4544), 1% (v/v) chemically defined lipid concentrate (Gibco 11905‐031), 10 mM HEPES (Gibco 15630‐056), 5% (v/v) fetal bovine serum (FBS, Bodinco, 5010), 100 units/ml of penicillin, and 100 μg/ml streptomycin at 37°C in a humidified atmosphere with 5% CO_2_ in tissue culture flasks precoated with 150 μg/ml rat tail collagen type‐I (Enzo LifeSciences ALX‐522‐435). C17.2 murine NSCs were maintained in DMEM (Gibco, 41965‐039) supplemented with 10% FBS, 5% Horse Serum (Invitrogen, 26050‐088), and 1% Penicillin‐Streptomycin sulfate (Gibco, 15140‐122) at 37°C under 5% CO_2_. The exosome donor cell line XP‐mCherry was generated by transfecting C17.2 cells with pCMV‐XP‐mCherry‐EF1‐Puro by electroporation performed in Amaxa 4D nucleofection system (Lonza) using SG transfection solution and program DN100 following manufacturer's instructions. A stable cell line was generated under antibiotic selection using Puromycin (Sigma, P8833, 3 µg/ml).

### 2.4 Preparation of exosome‐depleted Medium

To make exosome‐depleted FBS, FBS was diluted in DMEM (10%) and centrifuged at 110,000 *g* for 16 h at 4°C. The supernatant was then sterilized by passing through a 0.2‐μm filter (Millipore) and stored at 4°C.

### 2.5 Exosome isolation

pCMV‐XP‐mCherry‐EF1‐Puro‐expressing C17.2 cells were seeded in T162 flasks (Corning). Medium was replaced with exosome‐depleted medium when the cells reached ~40% confluency. After an incubation time of 48 hours, the medium was collected. Exosomes were isolated following a standard ultracentrifugation protocol (Thery et al., 2006). Briefly, cells and cellular debris were removed from the supernatant by centrifugation at 500 g and 2,000 g for 10 min, respectively (Beckman Coulter, Allegra X‐15R). Apoptotic vesicles and micro vesicles were removed by centrifugation at 10,000 g for 30 min (Sorvall Discovery 90SE ultracentrifuge, Beckman SW32i rotor). The resultant supernatant was subjected to ultracentrifugation at 110,000 g for 70 min to pellet down the exosomes (Beckman SW32i rotor). The pellet was resuspended in PBS and centrifuged again at the same conditions to obtain the final exosome pellet. The exosomes were resuspended in 50 µL PBS and the protein concentration was measured with DC protein assay kit (Bio‐Rad, 5000114).

### 2.6 Exosome characterization

The size, heterogeneity (polydispersity index), and surface charge (ζ‐potential) of the isolated exosomes were determined at RT with a Zetasizer Nano ZS particle analyzer using a DTS1070C capillary cell (Malvern, Worcestershire, United Kingdom) and a standard 633 nm laser, following the manufacturer's protocol. For quality assessment, 30 µg of exosomes or whole cell lysate was loaded onto an SDS‐PAGE gel and transferred to a PVDF membrane (Millipore, IPFL00010) at 500 mA for 70 minutes. The blot was blocked with Odyssey blocking buffer (Li‐COR, 927‐40000) for 1 hour at RT followed by primary antibody incubation (prepared in the blocking buffer) overnight at 4°C. The next day, blots were washed with 0.1% PBS‐Tween20 and incubated with secondary antibody solution (prepared in the blocking buffer) for 1 hour at RT. After washing with 0.1% PBS‐Tween20, the protein bands on the blot were visualized with an Odyssey® Infrared Imaging System (Li‐COR).

### 2.7 Exosome labeling with DiI

Exosomes were labeled by incorporating a lipophilic dye DiI (Invitrogen, D282) in exosome membranes. This was achieved by incubating purified exosomes with 1 µM DiI solution in PBS for 5 min at RT. The reaction mixture was ultracentrifuged at 100,000 g for 70 min at 4°C and excess DiI was removed by washing with PBS. The pellet, now containing DiI‐labeled exosomes, was resuspended in PBS and protein content was measured using DC protein assay kit.

### 2.8 Transport assay with DiI‐labeled exosomes in an in vitro transwell BBB model

50 × 10^3^/cm^2^ hCMEC/D3 cells were seeded on a transwell filter (Greinier, 665,641) precoated with 150 μg/ml rat tail collagen type‐I and grown for 5 days to confluency. Culture medium was replaced every other day, as described previously (De Jong et al., 2018). On the fifth day, the basolateral medium was replaced with 1 ml of pre‐warmed EBM‐2, and 500 µl EBM‐2 containing 20 µg/ml DiI‐labeled exosomes was added to the apical compartment. After incubation for 18 hours at 37°C, the apical and basolateral media were collected. The filters with cells were cut out and soaked in 1 ml water for 5 minutes. Apical, basolateral, and cellular fractions were transferred into black flat‐bottomed microplates (Greiner Bio‐One 655209) in triplicate and fluorescence intensities were quantified using a Fluostar‐Optima microplate reader (BMG Labtech) with 485 nm excitation wavelength and 520 nm emission wavelength, respectively. After subtracting the respective background fluorescence (serum‐free medium for apical and basolateral and water for cellular fractions), the percentage fluorescence associated with the apical, cellular, and basolateral fractions was calculated relative to the total fluorescent content of the apical, basolateral, and cellular fractions together.

### 2.9 Measurement of exosome transport in an in vitro transwell BBB model using dot blotting

hCMEC/D3 cell monolayers grown on Transwell filters were incubated with 500 µl EBM‐2 containing 20 µg/ml XP‐mCherry or wild‐type exosomes, in triplicate. After 18‐hour incubation, the medium in the apical compartments was collected and pooled. Similarly, the medium in the basal compartments was collected and pooled. Subsequently, the pooled media were subjected to ultracentrifugation at 110,000 *g* for 70 min at 4°C. Supernatants were discarded and pellets were resuspended in 5 µl PBS and blotted on a nitrocellulose membrane, air‐dried, blocked for 1 hour in Odyssey blocking buffer (Li‐COR biosciences, USA): 1x PBS (1:1) at RT and incubated overnight with anti‐mCherry antibody (Rabbit) in blocking buffer at 4°C. The next morning, the membranes were washed using 1x PBS/Tween‐20 (0.1%) and incubated with anti‐Rabbit secondary antibody conjugated to IRDye 800CW in blocking buffer for 1 hour at RT. Thereafter, membranes were washed with 1x PBS/Tween‐20 (0.1%), and directly imaged with an Odyssey Imaging system. Immunostaining signal intensities in the images were quantified using ImageJ. After subtracting the background signal intensity (wild‐type exosomes), the percentage intensity for apical and basolateral samples was calculated relative to the total signal intensity associated with the two samples.

### 2.10 Determination of mCherry orientation in exosomes via dot blotting

Exosomes, 0.25; 0.5; 1.0, and 2.0 µg in a volume of 2 µl, were blotted on a nitrocellulose membrane in duplo followed by drying. Then, the membranes were washed in PBS. Next, membranes were blocked with Odyssey blocking buffer and incubated with anti‐mCherry antibody (in blocking buffer) with or without Tween‐20 (0.1%). Lastly, anti‐Rabbit Odyssey secondary antibody conjugated to IRDye 800CW in blocking buffer was incubated with the membranes for 1 hour at RT, washed with 1x PBS/Tween‐20 (0.1%) and membranes were imaged with Odyssey imaging system.

### 2.11 Interaction of exosomes with hCMEC/D3 cells in the presence of inhibitors of endocytosis

5 × 10^4^/cm^2^ hCMEC/D3 cells (passage <38) were seeded on glass cover slips (VWR, 631‐1846) in a 24‐well plate precoated with 150 μg/ml rat tail collagen type‐I. Cells were grown for 5 days to confluency with medium replacement every other day. On the day of the experiment, cell monolayers were washed once with HBSS and incubated for 2 hours with 20 µg/ml DiI‐labeled exosomes in the absence or presence of metabolic inhibitors of endocytosis following pretreatment with just the inhibitors for 30 min, as previously described (Georgieva et al., 2011; Rejman et al., 2004; Rehman et al., 2011). Dimethylamiloride (DMA, 40 µM) was used to inhibit macropinocytosis, and Dynasore (Dyn, 80 µM) was used as an inhibitor of dynamin‐dependent endocytosis (blocking both clathrin‐ and caveolin‐mediated endocytosis). Similarly, cell monolayers were incubated with exosomes in the absence or presence of heparin (Sigma, H3393; 1, 10, and 50 µg/ml) and Heparinase III (HSase, Sigma, H8891; 50, 75, and 100 U/mL). Post incubation, cells were fixed with 4% paraformaldehyde, washed, and mounted on glass slides using Faramount mounting medium. The experiments were done three times in duplicate. Five random fields were imaged using a Leica DMI 6000B fluorescence microscope (HCX PL FLUOTAR L, 40x, NA 0.60 dry; using excitation/emission wavelengths 360/460 nm for DAPI and 550/570 nm for DiI). The number of fluorescent spots, representing exosome‐positive endosomes, per cell was quantified using ImageJ software (National Institutes of Health, http:// rsb.info.nih.gov/ij) using particle analysis and cell counter plugin (Schindelin et al., 2012).

For immunocytochemistry, cells were fixed with 4% PFA for 30 min at RT, washed with PBS and permeabilized with 0.2% Tween‐20 (Sigma, P1379) in PBS. After washing with PBS, they were incubated for 1 hour at RT with the blocking buffer (3% BSA (Sigma, A7906) in PBS). Anti‐SDC2 primary antibody prepared in blocking buffer was added to cells and incubated overnight at 4°C. Next, cells were washed with PBS thoroughly and incubated with secondary antibody in the blocking buffer and DAPI in blocking solution for 30 min at RT. After thoroughly washing with PBS, the coverslips were mounted on glass slides using Faramount mounting medium and imaged with confocal microscopy using Leica SP8 (HC PL APO CS2 63X, NA 1.4, oil immersion and excitation/emission wavelengths of 490/544 nm for GFP, and 358/463 nm for DAPI).

### 2.12 Statistical analysis

Statistical analysis was performed using two‐tailed Student's t‐test and ANOVA Tukey's post hoc test. Significant differences are indicated with *p < 0.05, **p < 0.01, ***p < 0.001, ****p < 0.0001. GraphPad Prism version 8 was used for all statistical analyses.

## 3 RESULTS

### 3.1 Characterization of C17.2 NSC exosomes

**FIGURE 1:**
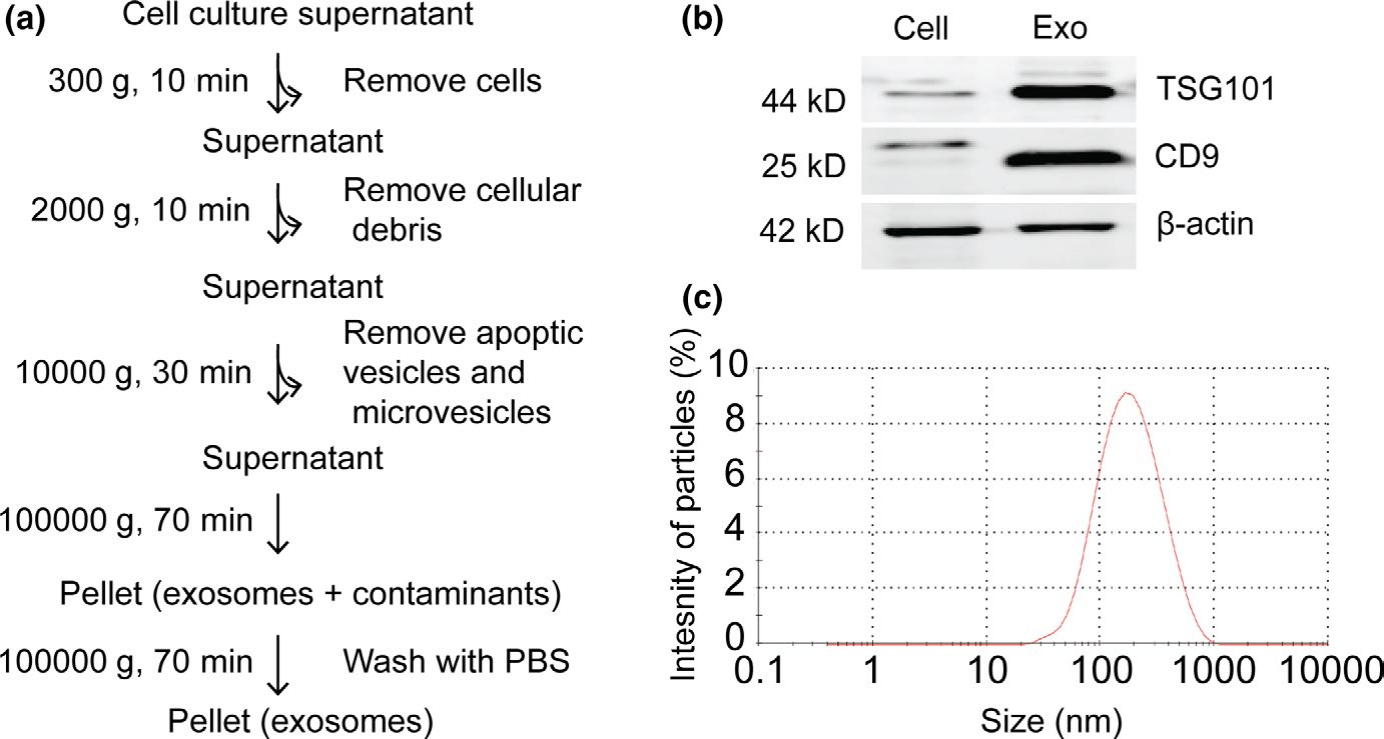
Characterization of exosomes derived from C17.2 neural stem cells. (a) Methodology used for exosome isolation. Cell culture supernatant 48 hours post seeding of C17.2 neural stem cells is collected and subjected to a series of centrifugations at different speeds to get rid of contaminants. In the end, high‐speed ultracentrifugation is used to collect exosomes. (b) Western blotting analysis of the exosome fraction obtained from procedure in (a) using exosome markers TSG101 and CD9. ß‐actin is used as a loading control. Cell and Exo correspond to parent cell and exosome lysates, respectively. 30 μg protein was loaded for both conditions. Exosome markers are enriched in the exosome fraction compared to the parent cells, while the ß‐actin amount is slightly lower in the exosome fraction. (c) Size distribution of exosomes measured by dynamic light scattering. Exosomes show a size of approximately 120 nm

To test the hypothesis that C17.2 NSC exosomes, similar to their parent cells, cross an in vitro BBB, we collected exosomes from NSCs by sequential centrifugation (Figure 1a). Different centrifugation speeds were employed to remove cells, debris, and microvesicles to isolate exosomes (Thery et al., 2006). We assessed the purity of our isolation by western blotting using antibodies against the exosome markers TSG101 (cytosolic protein related to multivesicular body (MVB) biogenesis (Colombo et al., 2014)) and CD9 (tetraspanin protein present in MVB membranes (Colombo et al., 2014)). Both proteins were enriched in the exosome fraction as compared to the parent cells. β‐actin was present in the exosome fraction in a lower amount compared to their parent cells (Figure 1b). Next, we characterized the exosome size distribution by dynamic light scattering (DLS) (Figure 1c). The exosomes were heterogeneous in size and had a diameter of 118.6 ± 14.5 nm with polydispersity index of 0.26 ± 0.02 and showed a negative ζ‐potential of −9.11 ± 4 mV in PBS (Table 1). Collectively, the C17.2 NSC‐derived vesicles that were isolated through sequential centrifugation showed phenotypical and physicochemical characteristics specific to exosomes.

**TABLE 1:** Size, PDI, and ζ ‐potential of unlabeled (Exo) and DiI‐labeled exosomes (Exo‐DiI) derived from C17.2 NSCs

Preparation	Size (nm)	Polydispersity index	ζ ‐potential (mV)
Exo	118.6 ± 14.5	0.26 ± 0.02	−9.11 ± 4
Exo‐DiI	142 ± 15.3	0.28 ± 0.03	−12.4 ± 0.65

Note

Three independent exosome isolations were subjected to DLS measurements.

### 3.2 C17.2 NSC exosomes cross an in vitro transwell model of the BBB

**FIGURE 2:**
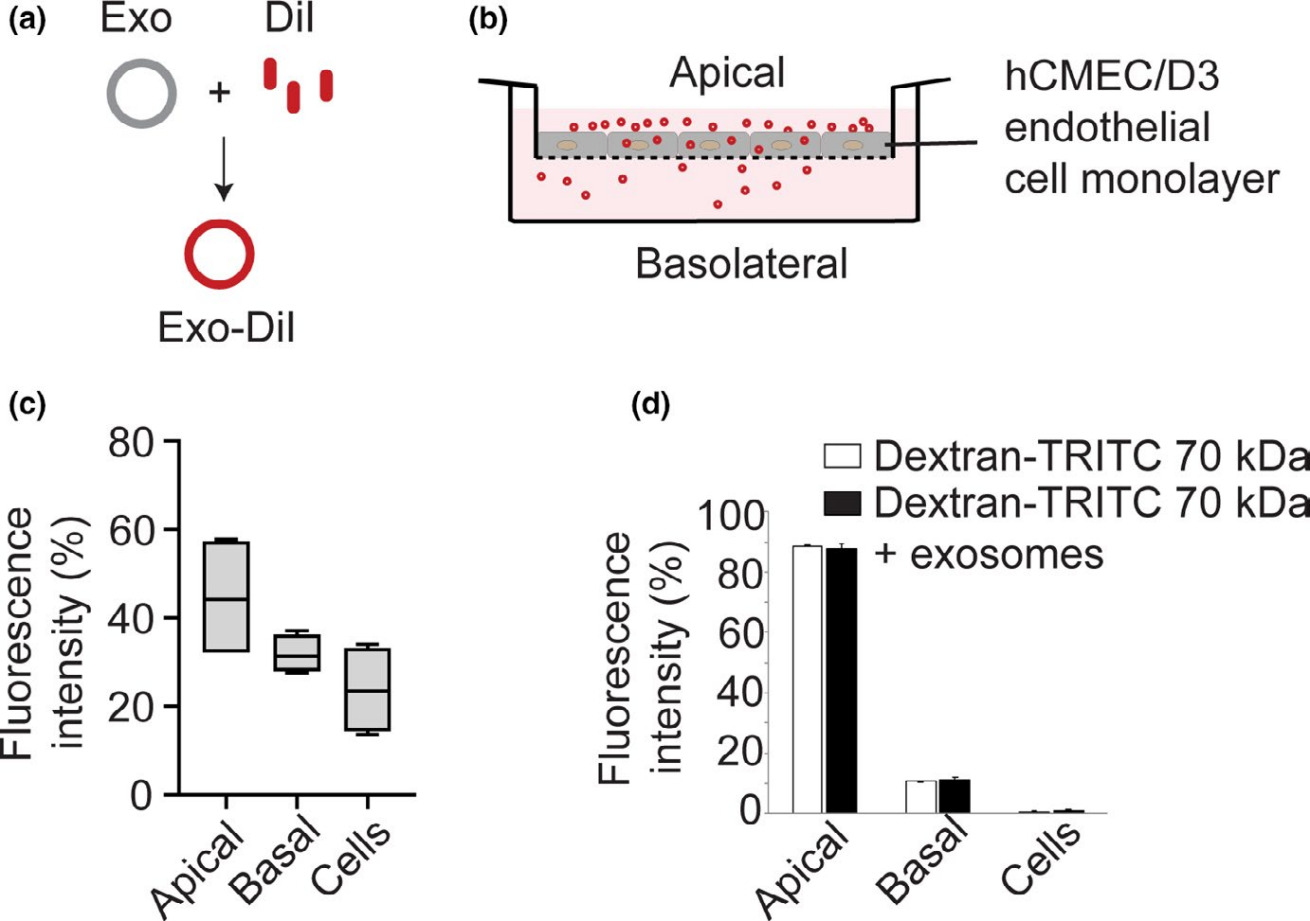
Transport of DiI‐labeled exosomes across an in vitro BBB model. (a) Cartoon depicting the spontaneous incorporation of the lipophilic DiI into the exosome membrane, generating DiI‐labeled exosomes (Exo‐DiI). (b) Schematic representation of the In vitro BBB model, composed of a hCMEC/D3 cell monolayer grown on a Transwell filter, incubated with DiI‐labeled exosomes. (c) Quantification of transcytosis of DiI labeled exosomes across the BBB model. 10 μg Exo‐DiI was added apically and incubated with the BBB model for 18 hours at 370C. DiI fluorescence associated with the apical, basolateral and cellular fractions was measured and is expressed relative to the combined DiI fluorescence of the three fractions (mean ± *SD*, *n* = 3). (d) Quantification of the paracellular permeability for 70 kDa dextran‐TRITC in the BBB model in the absence and presence of exosomes, to assess the integrity of the endothelial monolayer. Note that the endothelial monolayer integrity is not altered upon incubation with exosomes (mean ± *SD*, *n* = 3)

To quantify the transport of C17.2 NSC‐derived exosomes across an in vitro BBB, the exosomes were fluorescently labeled with DiI (Figure 2a). DiI incorporation increased the exosome size by ~23 nm (142 ± 15.3 nm) and decreased the ζ‐potential by ~2 mV (−12.4 ± 0.65 mV) as compared to unlabeled exosomes, without greatly affecting the polydispersity index (0.28 ± 0.03) (Table 1). DiI‐labeled exosomes (Exo‐DiI) were added to the apical side of the in vitro BBB model and incubated for 18 hours at 37°C (Figure 2b). 31.8 ± 5% of the exosomes reached the basolateral compartment, while 23 ± 10% was found within the hCMEC/D3 cells (Figure 2c).

To exclude an involvement of paracellular leakage due to disturbance of the endothelial monolayer integrity upon incubation with exosomes, a paracellular leakage assay was performed. To this end, the in vitro BBB model was incubated with TRITC‐labeled 70 kDa Dextran in the presence or absence of exosomes. Paracellular leakage of fluorescently labeled dextran was less than 10% in both conditions (Figure 2d), showing that the presence of exosomes did not significantly alter endothelial monolayer integrity. This suggests that C17.2 NSC exosomes efficiently translocate across the in vitro BBB model via transcellular transport. However, because DiI has a weak tendency to spontaneously exchange with cellular membranes (Daubeuf et al., 2009), some of the dye that is incorporated in exosomes may exchange with the endothelial cell membrane during exosome transport. As a consequence, the fluorescence signal from the basolateral compartment may have (partially) originated from basolaterally secreted hCMEC/D3 cell‐derived vesicles and/or debris, leading to an overestimation of the transcellular transport of the exosomes. To prevent exchange of fluorescence between exosomes and cell membranes, as could occur with exosomes post‐labeled with DiI, exosomes were labeled with a non‐exchangeable fluorescent protein through the expression of an XPack‐mCherry fusion protein in C17.2 parent cells, as described in the methods.

### 3.3 C17.2 NSC exosomes carry protein cargo across an in vitro BBB model

**FIGURE 3:**
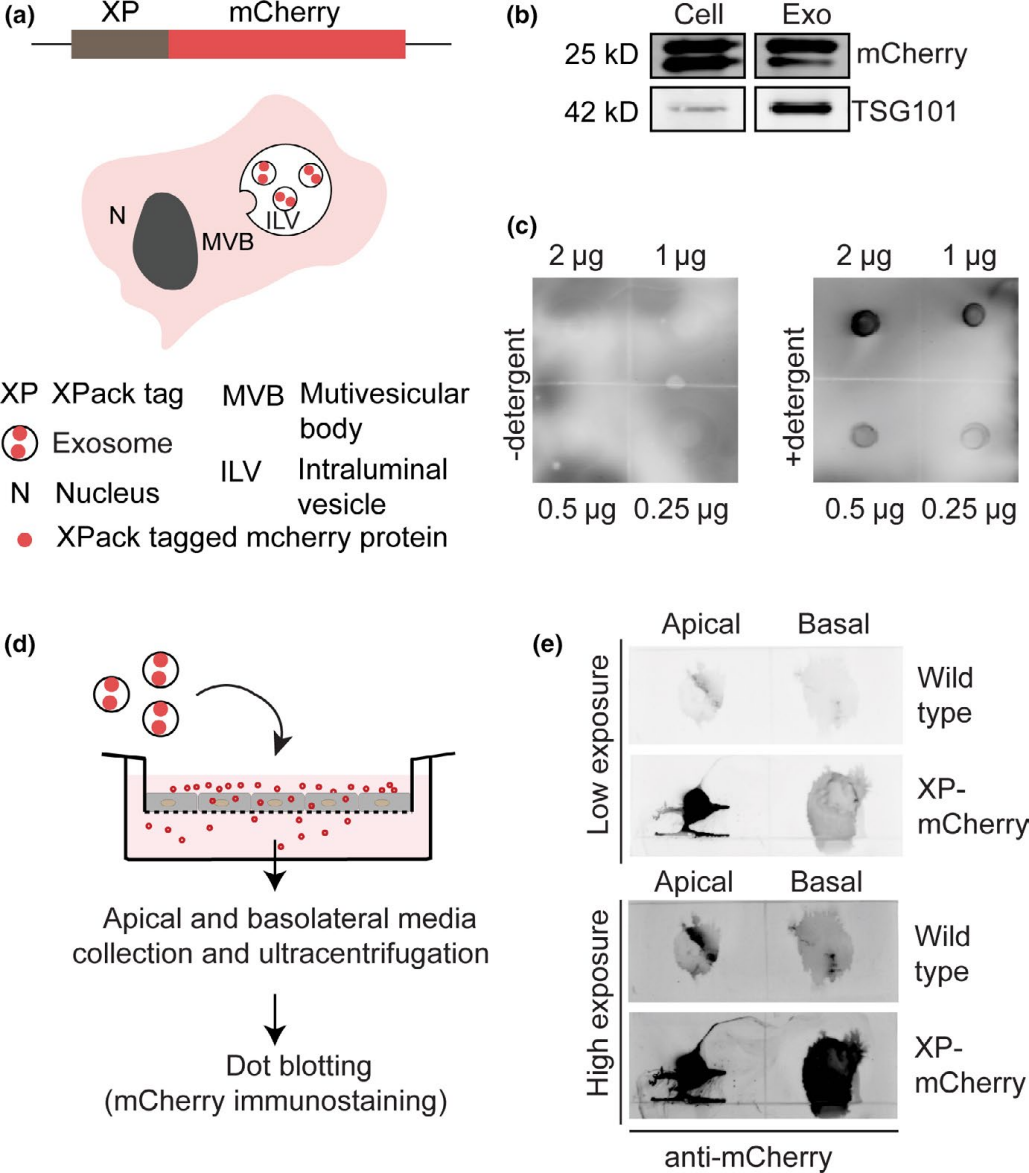
Transport of mCherry‐containing exosomes across an in vitro BBB model. (a) Schematic representation of the XP‐mCherry construct used in this study. Proteins tagged with the XP peptide are expressed at the cytosolic side of the plasma membrane and become concentrated in exosomes as they localize to multivesicular bodies, where ILVs (future exosomes) are generated. (b) Western blots of cell and exosome lysates show that XP‐mCherry is present in cells and exosomes. Exosome marker TSG101 is enriched in exosome fractions. (c) Dot blots of permeabilized (+detergent) and non‐permeabilized (−detergent) exosomes, demonstrating that XP‐mCherry is present at the exosome interior. Exosomes were dot‐blotted on nitrocellulose membrane in different quantities followed by anti‐mCherry immunostaining, in presence or absence of a detergent. (d) Schematic representation of the transcytosis assay. Exosomes containing XP‐mCherry are added to the apical compartment. After 18 hours, apical and basal media are collected and ultracentrifuged to collect exosomes. Collected exosomes are permeabilized and dot‐blotted, followed by mCherry immunostaining. (e) Dot blots of apical and basal fractions obtained from the in vitro BBB model after incubation with wildtype exosomes and XP‐mCherry exosomes, demonstrating XP‐mCherry signal in the basal fraction, which indicates effective transport of exosomes across the in vitro BBB model. The experiment was performed in triplicate, with apical fractions collected and pooled and basolateral fractions collected and pooled in order to obtain a detectable signal after dot blotting. XP: XPack; Exo: exosome lysate; Cell: whole cell lysate

To investigate the ability of exosomes to carry luminal cargo across the BBB, exosomes were loaded with mCherry protein using the commercial XP protein packaging system. In short, C17.2 cells were genetically engineered to stably express XP‐mCherry (Figure 3a). The XP tag enables active loading of mCherry into exosomes by directing the protein to the cytosolic side of the plasma membrane, which ends up at the cytosolic side of the MVB membrane and, subsequently, the luminal side of the exosomes (Shen et al., 2011; Yang & Gould, 2013). Western blotting of lysates of C17.2 XP‐mCherry‐expressing cells and their secreted exosomes revealed the presence of XP‐mCherry in both cells and exosomes, confirming the loading of XP tagged mCherry in exosomes. Moreover, TSG101 was enriched in XP‐mCherry exosomes compared to parent cells (Figure 3b), similar to in non‐labeled exosomes and their parent cells (Figure 1b). Next, the orientation of mCherry protein in the exosome membrane was verified by immunostaining of permeabilized and non‐permeabilized exosomes with anti‐mCherry antibody. Since XP‐mCherry is present within the lumen of the exosomes, mCherry antibody labeling should occur only in the presence of a detergent, that is, in permeabilized exosomes. Indeed, dot blotting of exosomes revealed that mCherry signal was detected only in permeabilized exosomes and was absent in non‐permeabilized exosomes (Figure 3c).

Next, a transport assay was performed with C17.2 wild‐type and XP‐mCherry exosomes. The exosomes were added to the apical compartment of an in vitro BBB model and after 18‐hour incubation, the apical and basolateral media were collected and ultracentrifuged to collect exosomes. The apical and basolateral fractions were then dot blotted to check for the presence of mCherry protein (Figure 3d). Upon incubation of the in vitro BBB with XP‐mCherry exosomes, both apical and basolateral fractions revealed mCherry signal, indicating the presence of exosomes (Figure 3e). In contrast, incubation with wild‐type (mCherry‐negative) exosomes resulted in both fractions being devoid of mCherry signal, as expected (Figure 3e). Dot blot signal analysis with ImageJ revealed that 38.4% of XP‐mCherry signal from not cell‐associated exosomes was retrieved from the basolateral compartment while 61.6% remained at the apical side. Collectively, the data show that NSC exosomes are capable of ferrying luminal cargo across the in vitro BBB.

Because DiI can spontaneously exchange between membranes, (part of) the basal DiI signal after incubation of the in vitro BBB model with Exo‐DiI may come from basally secreted hCMEC/D3‐derived membranes that have incorporated DiI from Exo‐DiI. On the contrary, XP‐mCherry protein cannot spontaneously exchange between cellular membranes. Therefore, the detection of mCherry signal directly reflects the presence of XP‐mCherry exosomes. In conclusion, to study exosome–cell interactions, the fluorescent labeling of exosomes by loading the exosomal lumen with fluorescent proteins through the genetic engineering of exosome producer cells may be preferred over the nearly effortless way of exosome labeling with lipophilic dyes.

### 3.4 Exosomes enter brain endothelial cells via endocytosis

**FIGURE 4:**
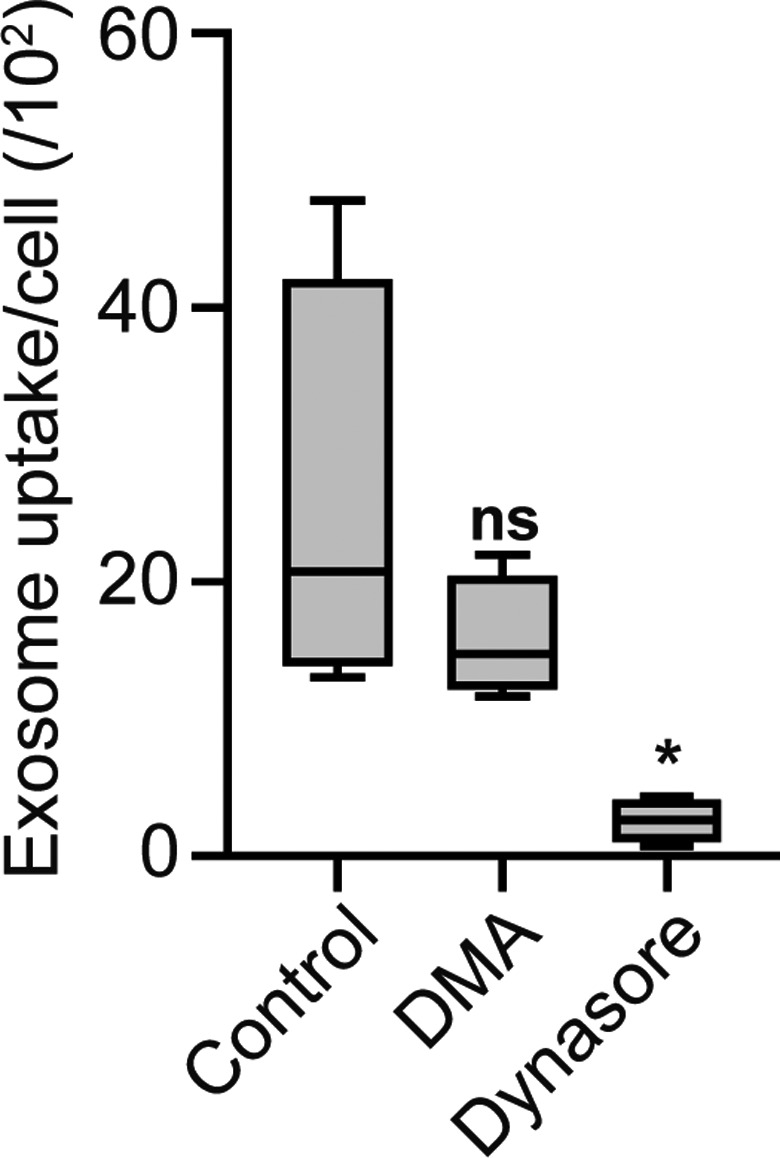
Endothelial cells internalize exosomes via dynamin‐dependent endocytosis. hCMEC/D3 cell monolayers were pre‐incubated with DMA (macropinocytosis inhibitor) or Dynasore (dynamin inhibitor) for 30 minutes at 37°C followed by incubation of exosomes in the continued presence of the inhibitor for 2 hours. Graph shows the relative number of exosomes per cell following incubation with Exo‐DiI in the absence (control) or presence of inhibitor. Exosome uptake in hCMEC/D3 cells is significantly reduced in presence of Dynasore (*n* = 4; ≥300 cells analyzed, *p < 0.05, ANOVA Tukey's post hoc test)

Paracellular transport of exosomes across the BBB seems unlikely when taking into account the size limit for paracellular transport of molecules, that is 500 Da, and the relatively large size of the exosomes, that is, 118.6 ± 14.5 nm (see Table 1). In addition, the presence of exosomes did not enhance the paracellular leakage of 70 kDa dextran, which has a hydrodynamic radius of <10 nm (Figure 2d). Thus, we next investigated the involvement of endocytosis in exosome internalization by brain endothelial cells. For this purpose, hCMEC/D3 cell monolayers were incubated with exosomes in the absence and presence of metabolic inhibitors of endocytosis, specifically DMA and dynasore, inhibitors of macropinocytosis, and dynamin‐dependent endocytosis, respectively (Georgieva et al., 2011; Rejman et al., 2004; Rehman et al., 2011). While macropinocytosis generally is a dynamin‐independent process, dynasore interferes with dynamin GTPase activity and is known to affect both clathrin‐ and caveolin‐mediated endocytosis (Macia et al., 2006). In the presence of dynasore a significant, nearly complete inhibition of exosome uptake in hCMEC/D3 cells was observed, whereas DMA was without effect (Figure 4), suggesting that exosome uptake involves dynamin‐dependent endocytosis.

### 3.5 Exosomes interact with HSPGs to enter brain endothelial cells

**FIGURE 5:**
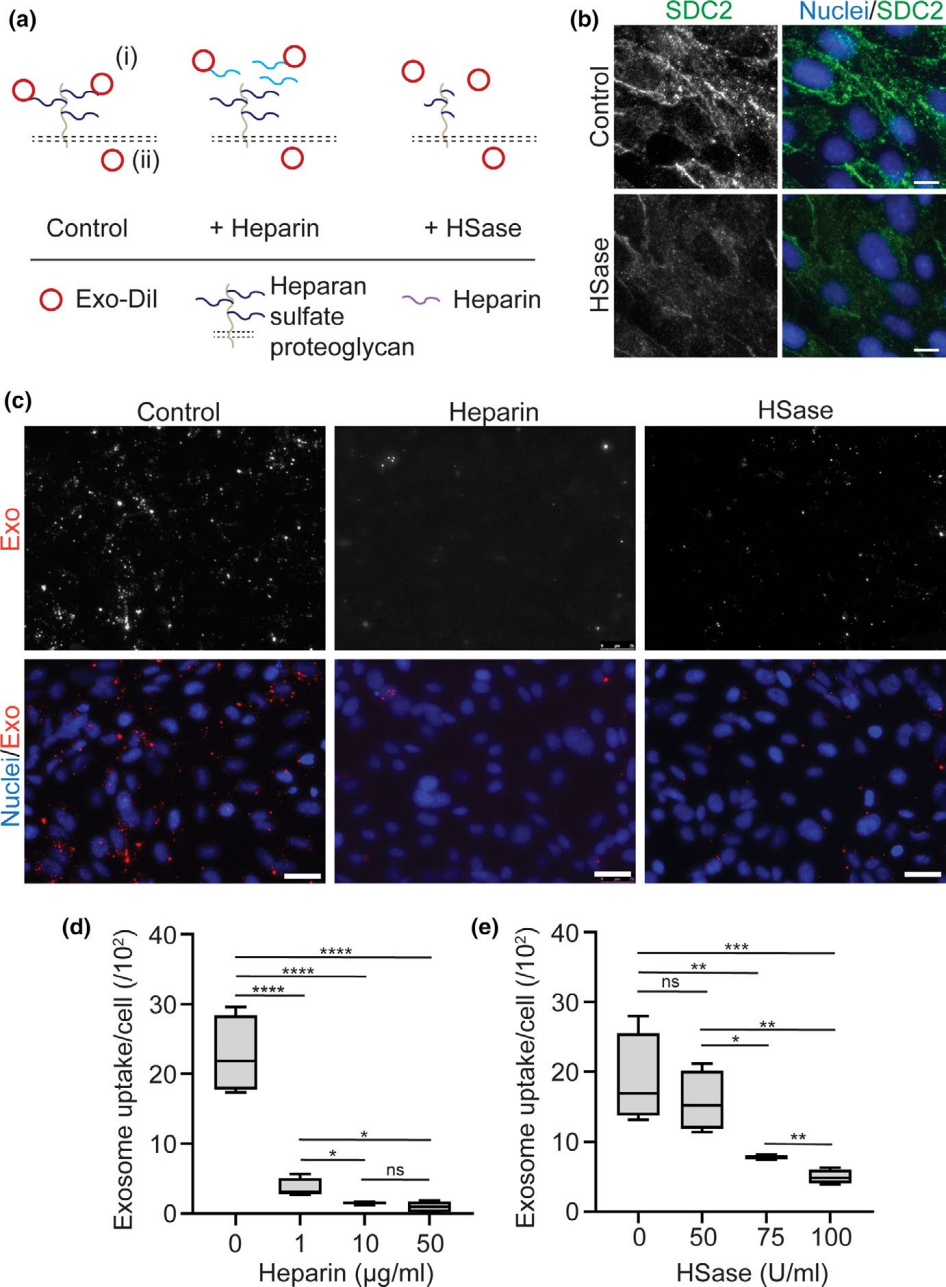
Exosomes interact with HSPGs in hCMEC/D3 cells. (a) Schematic representation of the possible effects of heparin and HSase on the interaction of exosomes with hCMEC/D3 cells. (b) SDC2 antibodystaining for assessing the effect of HSase to remove HSPGs enzymatically. Note that SDC2 immunostaining signal is almost absent in cells treated with HSase. Scale bar = 10 μm. (c) Fluorescence images of hCMEC/D3 images incubated with Exo‐DiI in absence (control) or presence of 50 μg/ml heparin or 100 U/ml HSase. Exosome interaction with hCMEC/D3 cells is nearly abolished in presence of heparin and HSase. Scale bar = 25 μm. (d) Quantification of Exo‐DiI uptake in hCMEC/D3 cells in absence (control) or presence of 1, 10 or 50 μg/ml heparin (*n* = 4; ≥300 cells analyzed per time point, *p < 0.05, ****P < 0.0001, ns – nonsignificant, ANOVA Tukey's post hoc test for comparison of each treatment condition with control, unpaired t‐test for comparison between treatment conditions). (e) Quantification of Exo‐DiI uptake in hCMEC/D3 cells in the absence (control) or presence of 50, 75 or 100 U/ml HSase (*n* = 4; ≥ 300 cells analyzed per time point, *p < 0.05, **p < 0.01, ***p < 0.001, ANOVA Tukey's post hoc test for comparison of each treatment condition with control, unpaired *t*‐test for comparison between treatment conditions)

Because endocytosis of nanoparticles generally involves cell surface receptors that mediate nanoparticle binding and/or uptake (Matsumoto et al., 2017; Rehman et al., 2012; Wang et al., 2016; Zuhorn et al., 2007), we next examined the possible role of cell surface receptors in the interaction between NSC exosomes and hCMEC/D3 cells. Heparan sulfate proteoglycans (HSPGs) are highly sulfated glycoproteins, containing one or more HS chains. They are present at the cell surface and extracellular matrix and interact with a myriad of ligands (Sarrazin et al., 2011; Lindahl et al., 2017). HSPGs are abundantly present in the brain endothelium (Bobardt et al., 2004; Vorbrodt, 1989) where they act as receptors for among others brain tropic viruses such as HIV (Argyris et al., 2003; Bobardt et al., 2004; Floris et al., 2003; Leupold et al., 2008; Qiao et al., 2003). Exosomes derived from cancer cells and hepatic stellate cells were also shown to interact with HSPGs preceding cellular uptake (Chen & Brigstock, 2016; Christianson et al., 2013). Hence, we investigated if HSPGs act as receptors for NSC exosomes in brain endothelial cells. To this end, two methods were employed, that is, competitive inhibition with free heparin, that is, an HS mimetic (Tefferi et al., 1989; Sarrazin et al., 2011; Shriver et al., 2012), and enzymatic degradation of heparan sulfates by Heparinase III (HSase). If HSPGs play a role in exosome uptake, incubation of hCMEC/D3 cell monolayers with exosomes in the presence of heparin or HSase would result in diminished exosome uptake as compared to incubation in the absence of heparin or HSase (Figure 5a). First, we checked for the presence of HSPGs in hCMEC/D3 cells. Syndecan‐2 (SDC2) is a type of HSPG abundantly present in endothelial cells (Floris et al., 2003). Indeed, immunostaining showed that SDC2 was abundantly present in the brain endothelial cells as was reported before (Floris et al., 2003) (Figure 5b). When cells were treated with HSase, SDC2 immunostaining was diminished, showing that HSase was effective in degrading HSPGs in our system (Figure 5b). Next, hCMEC/D3 cell monolayers were treated with Exo‐DiI in the presence or absence of heparin or HSase. Preincubation with heparin as well as HSase showed a significant decrease in exosome uptake (Figure 5c). Exosome uptake decreased in a dose‐dependent manner for both the heparin and HSase treatment (Figure 5d and e). Specifically, exosome uptake was reduced by 84 ± 4.8% in the presence of as low as 1 µg/ml heparin and further reduced by 93 ± 1.1% and 94 ± 2.6% in the presence of 10 and 50 µg/ml heparin, respectively (Figure 5d). Treatment of cells with 50 U/ml HSase led to 27 ± 6% inhibition of exosome uptake, while 100 U/ml inhibited exosome uptake by 79 ± 3% (Figure 5e). Taken together, our data show that HSPGs play an active role, presumably as binding receptors, in NSC exosome uptake by brain endothelial cells.

## 4 DISCUSSION

Drug delivery to the brain continues to be a challenge, hampering the development of treatments for brain disorders. Here, we show that exosomes derived from NSCs have the intrinsic capacity to cross an in vitro BBB consisting of human brain endothelial (hCMEC/D3) cells. hCMEC/D3 cells internalized NSC exosomes via dynamin‐dependent endocytosis. Importantly, we show that exosomes interact with the brain endothelial cells via HSPGs. Furthermore, exosomes were able to carry a protein cargo across the in vitro BBB, substantiating their potential as delivery vehicles to treat brain disorders. Taken together, our data encourage the development of exosomes as delivery vehicles for the treatment of brain disorders via intravenous administration, obviating the need for invasive intracerebral or intracerebroventricular administration routes. Moreover, active HSPG targeting of nanoparticles, including exosomes, may be exploited for effective crossing of the BBB.

Our data indicate that HSPGs on brain endothelial cells may play a crucial role in exosome uptake. The brain endothelial cell membrane is rich in HSPGs (Bobardt et al., 2004; Floris et al., 2003), and viruses, for example, HIV (Argyris et al., 2003; Bobardt et al., 2004; Jones et al., 2005), murine leukemia virus (Jinno‐Oue et al., 2001), and herpes simplex virus(Spear, 2004) interact with HSPGs to breach the BBB. Interestingly, HIV interacts with HSPGs primarily to transmigrate across the BBB while it binds another receptor to infect brain endothelial cells, suggesting that HSPGs may act specifically as receptors for transcytosis. Moreover, HSPGs can act as primary or secondary receptors (Sarrazin et al., 2011). Thus, other receptor(s) in addition to HSPGs may be essential for exosome uptake and transcytosis. Here, we show that HSPGs play a role in the uptake of C17.2 NSC exosomes in hCMEC/D3 cells. However, whether HSPGs act as true internalization receptors rather than attachment sites remains to be explored. Recently, adsorptive transcytosis was shown to play a role in exosome transport across the BBB (Banks et al., 2020), suggesting an involvement of electrostatic interaction between exosome membrane components and endothelial cell surface receptors (Villegas & Broadwell, 1993; Banks et al., 1997; Banks et al., 2020). In addition, HSPGs were shown to be essential for the uptake of and biological response to cancer exosomes in target cells (Christianson et al., 2013). Similarly, C17.2 NSC exosomes that do not transcytose could be used to evoke a biological, potentially therapeutic, response in brain endothelial cells.

Interestingly, while HSPGs represent a ubiquitous attachment site for various ligands (Sarrazin et al., 2011), their HS chain composition is cell type‐dependent (Allen & Rapraeger, 2003; Condomitti & de Wit, 2018; Xu & Esko, 2014). This means that specific HSPG ligands show cell type‐specific binding, which will affect their biodistribution upon systemic administration. Recently, 2‐O and N sulfates were shown to be necessary for binding of cancer exosomes to recipient cells (Christianson et al., 2013). Similarly, it would be of great interest to identify the specific sulfation pattern of HS on brain endothelial cells that mediates the binding of NSC exosomes, to develop brain‐specific HSPG‐targeted nanoparticles for the treatment of brain disorders.

Brain inflammation is a common condition associated with CNS disorders (Schain & Kreisl, 2017). Cells like NSCs, monocytes, and macrophages show a higher propensity to cross the BBB under such conditions (Bjugstad et al., 2005; Floris et al., 2003; Kelly et al., 2004; Yuan et al., 2017). Recently, macrophage‐derived exosomes were reported to cross the BBB in vitro and in vivo, under inflammatory conditions (Yuan et al., 2017). Whether exosomes derived from NSCs show similar capability remains unexplored. HSPGs act as a key regulator in facilitating and increasing extravasation of immune cells to sites of inflammation (Kumar et al., 2015). The fact that NSC exosomes interact with HSPGs for endothelial cell entry may point toward a potential inflammation‐responsive behavior of exosomes. NSCs interact with endothelial cells via CD44, VCAM‐1, and ICAM‐1 (Rampon et al., 2008). Similarly, exosomes derived from NSCs could also interact with these receptors for transcytosis. Although incubation with heparin and HSase led to near complete inhibition of NSC exosome uptake in hCMEC/D3 cells, HSPGs may act as binding receptors and other receptors such as VCAM‐1 and ICAM‐1 may be responsible for exosome uptake and transcytosis.

The use of drug‐loaded nanoparticles and drug conjugates to deliver therapeutic biomolecules to the brain has achieved limited success (Abbott & Romero, 1996; Banks, 2016; Razpotnik et al., 2017; Tang et al., 2019; Yu et al., 2014), mainly due to poor biodistribution. Exosomes have the potential to significantly improve the biodistribution as a result of their organotropic behavior (Antimisiaris et al., 2018; Hoshino et al., 2015; Wiklander et al., 2015). Moreover, exosomes pose less safety risks than synthetic delivery systems because of their non‐immunogenicity when derived from, for example, patient‐specific cell sources (Kim et al., 2005) or agricultural products, such as fruits (Antimisiaris et al., 2018; Colombo et al., 2014; Ju et al., 2013; Wang et al., 2015). In addition, as opposed to synthetic delivery platforms that carry just the therapeutic drug, exosomes carry additional cargo including proteins and miRNAs (Thery et al., 2002), which may confer treatment advantages. For example, exosomes are rich in GM1 ganglioside and cholesterol (Skotland et al., 2017), which have been shown to ameliorate Huntington's disease symptoms in vitro and in vivo (Alpaugh et al., 2017; Valenza et al., 2015). Inventively, NSCs have been engineered to continually secrete exosomes containing therapeutic cargo at a high dose using a booster plasmid, providing a continuous source of the therapeutic following their intracerebral implantation (Kojima et al., 2018). Along the same line, but preventing the use of stem cells in order to prevent possible erroneous differentiation, (brain) endothelial cells may be genetically engineered in vivo to generate therapeutic exosomes. Importantly, in depth knowledge of the biogenesis of exosomes and their natural content is needed to be able to evaluate the safety of exosome‐based therapeutics in a clinical setting.

Taken together, our findings show that NSC exosomes can be employed as drug delivery vehicles to cross the BBB. The elucidated HSPG‐dependent mechanism of their interaction with the BBB identifies a potentially targetable pathway for improving transcytosis of therapeutic molecules and/or drug delivery systems across the BBB.

## 5 COMPETING INTEREST

The authors declare no competing interests.

## AUTHORS’ CONTRIBUTIONS

BSJ and ISZ conceived the project. BSJ performed the experiments. All authors developed experimental design, performed analysis, and wrote and edited the manuscript.

## ETHICAL APPROVAL

The authors have read and adhered to the ethical standards statement for manuscripts submitted to the European Journal of Neuroscience.

### PEER REVIEW

The peer review history for this article is available at https://publons.com/publon/10.1111/ejn.14974

## Data Availability

Raw data files are available on request.
